# Exploring the interplay between guidance services and career success: Unveiling the key determinants?

**DOI:** 10.1371/journal.pone.0314200

**Published:** 2025-01-29

**Authors:** Paul Mensah Agyei, Joyce Kwakyewaa Dankyi, Vincent Mensah Minadzi, Lydia Aframea Dankyi, Moses Segbenya

**Affiliations:** 1 Department of Business Studies, College of Distance Education, University of Cape Coast, Cape Coast, Ghana; 2 Department of Education, College of Distance Education, University of Cape Coast, Cape Coast, Ghana; Ladoke Akintola University of Technology Teaching Hospital: LAUTECH Teaching Hospital, NIGERIA

## Abstract

This study examines the effect of determinants of stakeholders’ attitudes on the perceived relevance of guidance services, policy implementation, and career success. An explanatory research design was adopted for the study. A sample of 413 guidance service coordinators at the regional, district, and school levels were selected. A self-developed questionnaire was used for the data collection. It was found that the availability of funds (finance) for payment of expenses incurred in relation to guidance services, competent and adequate guidance coordinators, and a dedicated office to ensure confidentiality for guidance services were the three determinants that predicted the perceived relevance of guidance services in Ghana. Additionally, provisions for guidance services on the teaching timetable, availability of logistics (material resources), and quality guidance coordinators were the only three determinants that influenced policy implementation and monitoring of guidance services at the basic school levels in Ghana. Human resources again emerged as the only determinant that influenced the career success of beneficiaries in Ghana. The study recommends that Ghana Education Service and heads of basic schools should appoint competent and adequate guidance service coordinators, resource them financially and logistically, allocate guidance services on the teaching timetable, and provide a dedicated office to ensure confidentiality.

## Introduction

Guidance is a specialist field that offers a wide range of initiatives and services meant to assist individuals in understanding who they are, the difficulties they face, and the world around them [1]. Formal guidance services can be traced to the 1890s and 1900 in the USA. However, its foundation can be traced back to the works of Plato and Aristotle. Since then, the importance of guidance services to its target audience has not been challenged. Its importance in schools for that matter drawn the attention of scholars and stakeholders [2–4]. The effects of guidance services on individuals particularly the young ones who must be guided to make the right decision for a better future cannot be understated [5]. When guidance services are implemented well, it goes a long way to help young people to make the right choices thereby enhancing their career success.

Career success is at the heart of every professional. This requires a lot of investments in career development activities. One such investment is guidance services. A relationship between career success and guidance service has been established in the literature. [6] for instance, are convinced that guidance services boost individuals’ capacities such that they contribute to enhancing jobs, skills, and growth. Many factors account for learners’ choice of a particular career [7]. As such, the success of a chosen career is dependent on the successful evaluation of these factors. This brings to light the role of guidance services in career planning and success. Guidance service providers guide young people at the early stages in schools to evaluate and make informed and appropriate decisions regarding their respective career paths or choices.

The relevance of guidance service to career success requires the effort of all stakeholders in order to ensure its success [8]. Though lots of studies have been done on guidance services for students, parents, and classroom teachers [9], not much attention has been given to district and school guidance coordinators who play critical roles when it comes to policy implementation at the school level. It has been established that the effective delivery of service is highly dependent on the quality of human, capital, and material resources [2] and the effective implementation of strategies and policies. Besides, the role of time for the guidance service activities, human and material resources for policy implementation, and monitoring of guidance services are yet to be fully explored. The contribution of these variables in guidance service delivery has not received the required attention.

Existing studies [5, 2–4] have examined the influence of stakeholders’ attitudes on career success among beneficiaries of guidance services. However, specific components or determinants of stakeholders’ attitudes that influence career success, perceived relevance, and policy implementation and monitoring of guidance services in Ghanaian basic schools are yet to be investigated after sixty-six years of guidance service delivery in Ghana. Identification of these specific determinants of the attitude of stakeholders is necessary for policy direction to ensure that guidance services in Ghanaian basic schools benefit the targets. It will also contribute to the extant literature on effective guidance service delivery in first-cycle institutions. This study therefore fills the conceptual gap in the literature by examining the effect of determinants of stakeholders’ attitudes toward the perceived relevance of guidance services, policy implementation, and career success.

Empirical study by [3] suggests that the availability of facilities and resources has a significant influence on the effective delivery of guidance services in schools. It was revealed that facilities such as guidance reference books, career resource centers, and appropriate time allocated for guidance and counseling were lacking. The findings were consistent with the study by [10] that critical resources must be readily available for the successful running of guidance services in schools. Once these resources are not at hand, they would directly influence how guidance services are implemented, monitored, and evaluated in schools. By extension, if authorities and state institutions see guidance services to be relevant, they would provide the necessary logistics and materials.

Meanwhile, the study by [3] indicated that lack of funds was a challenge regarding guidance services in the studied schools. Their findings were in tandem with an earlier study in Nigeria by [11]. Lack of funds to a large extent means that guidance services in our schools especially at the basic level are considered to be irrelevant hence the inept attitudes towards it. A study by [12] investigating principals’ effective implementation of guidance services as a correlate to students’ moral behaviour in secondary schools showed that inadequate administrative manpower/lack of competent guidance coordinators was affecting guidance service delivery in the education system. This revelation was consistent with an earlier investigation by [2] that the majority of staff who handled guidance services were untrained leading to poor implementation of the services. The findings were consistent with the earlier work by [13] who indicated that apart from a lack of quality human resource, inadequate material resources such as office space, time allocation, and furniture were inhibiting guidance service delivery.

Implementation, monitoring, and evaluation of guidance services are key to realizing the full benefits thereof. Within this context, [2] undertook a study that found that there was a relationship between material resources and how guidance services are implemented, monitored, and evaluated. Their study revealed that the majority of the respondents did not patronize guidance services because of a lack of resources such as office space. By its nature, guidance services are undertaken in a serene and comfortable place devoid of noise and distraction. How can we effectively implement guidance services, let alone monitor and evaluate if there is no dedicated office space for it? Whether relevance and career success relate significantly to each other was one of the objectives of the current study. A study by [14] revealed that stakeholders (guidance coordinators, teachers, and parents) disagreed that there was a need to provide guidance services in the area of career choice in schools. This is revealing since stakeholders would have intimated that guidance services in the area of career choice be provided to students if it would go a long way to benefit students in their future vocations (career success). This suggests therefore that there is no relationship between career success and implementation of guidance services at the basic school levels.

Moreover, a study undertaken by [15] showed that there were challenges regarding the implementation of guidance services in the studied schools. The study indicated that there was a lack of time, funding, inadequate facilities, uncooperative clients, and qualified personnel were among the challenges facing the implementation of the guidance and counselling programme. The study concluded that guidance and counselling services were not implemented.

### Theoretical perspectives

**Fig 1:**
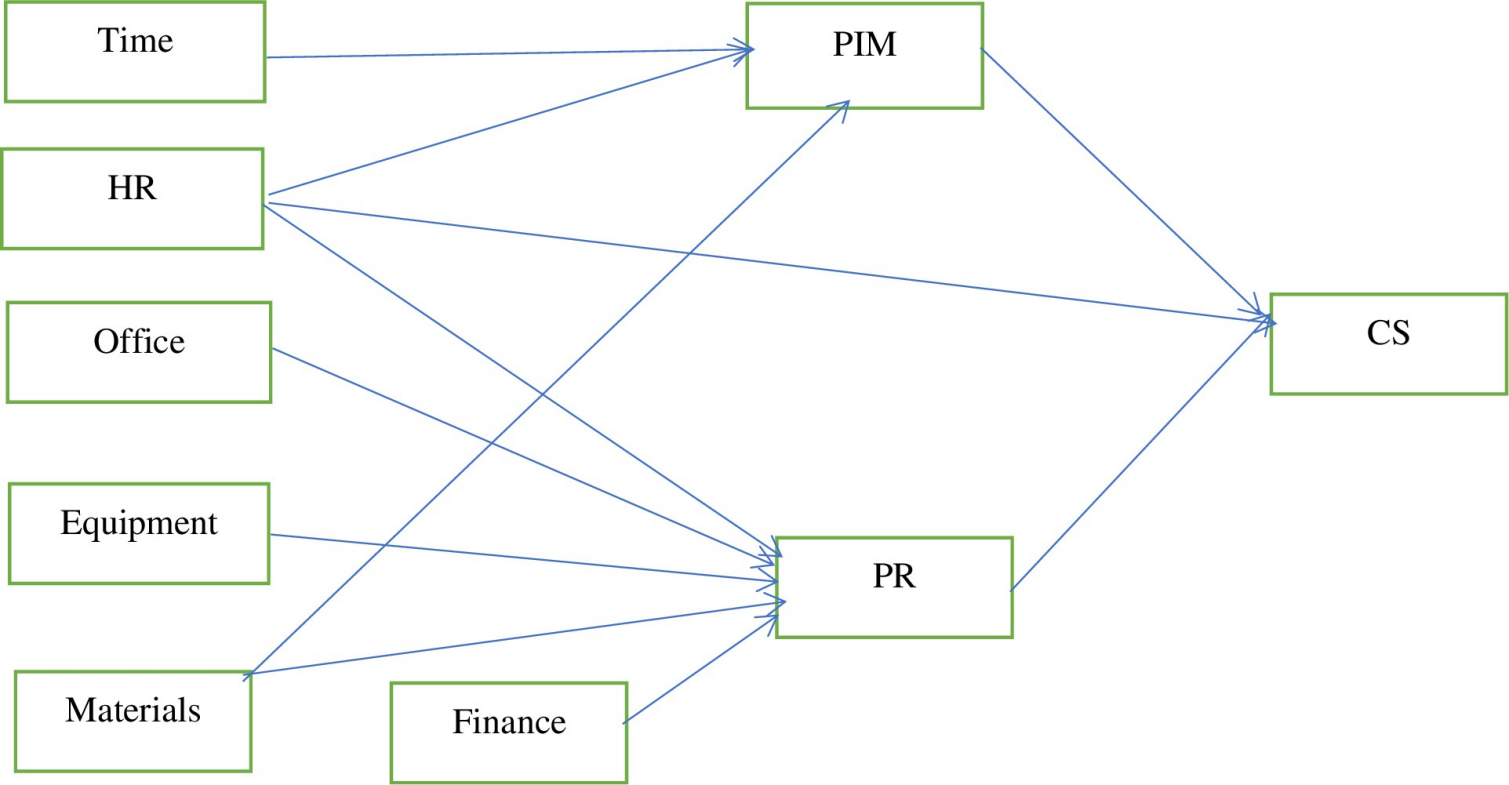
Conceptual framework guiding the study. **Key:** CS = career success, PR = perceived relevance of guidance service, PIM = policy implementation and monitoring on guidance service, HR = Human resource.

The Social Learning Theory (SLT) by John Krumboltz was chosen as the framework for providing students with guidance services in schools. This theory is premised on the assumptions that a specific combination of one’s genetic makeup and social environment determine the chances for attaining one’s goals, particularly, the ones that are related to academic and career opportunities [16]. This theory comprises four concepts namely genetic endowment, environmental factors, learning experiences and task approach skills [17]. For an individual to be successful in his or her future career, the four elements–the natural gifts, the environment, academic experience, and what the students can do (goal setting, value clarification, generating alternatives, predicting future events, and obtaining occupational information) must be considered when providing guidance services. The task skills would come as a result of interactions between the genetic endowment, environmental factors, and learning experiences that culminate in undertaking various tasks. This would enable the individual to match his/her vocation with interest. Even though the theory implies that one’s opportunities may be limited to one’s intrinsic abilities, it will also help guidance coordinators to focus on the areas of studying that students find engaging and in which they do well hence, determining the possibility of their career success. The rationale behind adopting Krumboltz’s SLT is that the framework would help guidance coordinators identify the strengths and weaknesses of students thereby determining the opportunities that would be available for them as far as their career success is concerned. For guidance service delivery to be effective, there must be conducive room or space and logistics, time allocation, funding, and above all, positive attitudes towards it by stakeholders. The conceptual framework carved to guide the study is provided in Fig 1.

Based on the review of the exact literature and the conceptual framework and with emphasis on Ghanaian basic schools, the study tests the following hypotheses:

H1: There is significant relationship between the availability of equipment and the perceived relevance of guidance services at the basic school level in Ghana.
H2: There is significant relationship between the availability of funds (Finance) and the perceived relevance of guidance services at the basic school level in Ghana.
H3: There is significant relationship between the availability of competent and adequate human resource and the perceived relevance of guidance services at the basic school level in Ghana.
H4: There is significant relationship between the availability of materials/logistics and perceived relevance of guidance services at the basic school level in Ghana.
H5: There is significant relationship between the availability of dedicated office space/complex and perceived relevance of guidance services at the basic school level in Ghana.
H6: There is significant relationship between the availability of competent and adequate human resource and policy implementation and monitoring of guidance services at the basic school level in Ghana.
H7: There is significant relationship between availability of allocation of time on teaching timetable for guidance services and policy implementation and monitoring on guidance services at the basic school level in Ghana.
H8: There is significant relationship between the availability of availability of materials/logistics and policy implementation and monitoring of guidance services at the basic school level in Ghana.
H9: There is significant relationship between the availability of competent and adequate human resource and career success among beneficiaries of guidance services at the basic school level in Ghana.
H10: There is significant relationship between policy implementation and monitoring on guidance services and career success among beneficiaries of guidance services at the basic school level in Ghana.
H11: There is significant relationship between the perceived relevance of guidance services and career success among beneficiaries of guidance services at the basic school level in Ghana.

## Materials and methods

The study sought to examine the effect of the determinants on stakeholders’ attitudes toward the perceived relevance of guidance services, policy implementation, and career success. Guided by quantitatively inclined objectives, we adopted the positivist paradigm approach and explanatory research design. With the aid of multistage sampling techniques, stratified and simple random sampling techniques, data was collected from 413 stakeholders of guidance services from a study population of 16,164 across fifteen administrative regions in Ghana. These were made up of district guidance coordinators, school guidance coordinators, and basic school headteachers. The sample provides a good representation (2.6 percent) of the study’s population. [18] sample determination table says that for a population of 16, 000, a sample size of 377 is best, but the sample size of 413 is more preferred.

Data collection was done from 1^st^ October, 2022 to 22^nd^ December, 2022. Data was obtained with the aid of a self-administered questionnaire. With the exception of demographic data, all variables were measured with a four-point Likert-type scale, such as strongly disagree, disagree, agree, and strongly agree. The instrument had five parts. The first part was dedicated to the respondents’ demographic characteristics. The second and third focused on stakeholders’ attitudes towards guidance services and policy implementation, monitoring, and evaluation of guidance services. Part four addressed the relevance of guidance services, whilst the last part was dedicated to career success. With a pre-test reliability Cronbach alpha value above 0.70, the instrument became appropriate for the main data collection after a few modifications [19]. The study research questions were assessed with a Partial Least Square-Structural Equation Modelling (PLS-SEM). The reliability and validity checks proved that the study is valid and reliable (see, Tables 1 and 2) [20]. The study met all ethical concerns (e.g., informed consent, freedom to participate and withdraw at any time during the study, no harm to the research participant, confidentiality, and anonymity). Again, the researchers received an ethical clearance certificate from the Institutional Review Board (ID—UCCIRB/EXT/2022/24) of the University of Cape Coast.

**Table 1:** Demographic characteristics.

Demographic characteristics	Frequency	Percent
Stakeholder		
School headteachers	83	20.1
School Guidance Coordinators	207	50.1
District Guidance coordinators	123	29.8
Total	413	100.0
**Tenure**		
1–5 Years	87	21.1
6–10 Years	146	35.4
11 years and above	180	43.6
Total	413	100.0
**Gender of respondents**		
Male	157	38.0
Female	256	62.0
Total	413	100.0

Source: Field Survey (2022)

**Table 2:** Construct reliability and validity.

	Cronbch’s Alpha	rho_A	Composite Reliability	Average Variance Extracted (AVE)
CS	0.920	0.945	0.934	0.638
Equipment	0.951	0.953	0.965	0.873
Finance	0.883	0.884	0.928	0.811
HR	0.387	0.737	0.410	0.655
Materials	0.878	0.884	0.926	0.806
Office	0.846	0.870	0.906	0.762
PIM	0.893	0.896	0.917	0.615
PR	0.969	0.973	0.973	0.781
Time	0.843	0.854	0.906	0.763

Source: Field Survey (2022)

## Results

Table 1 presents results for the demographic characteristics of respondents. The results suggest that the majority of the respondents were school guidance coordinators (50.1%), had worked for 11 years and above (43.6%), and were female respondents (62.0%).

### Measurement of the structural model

**Fig 2:**
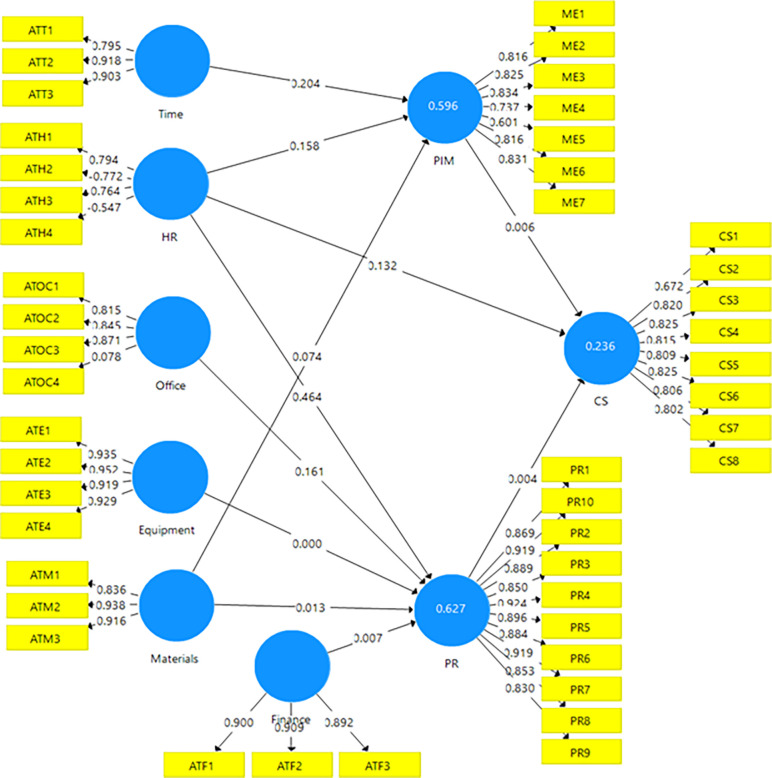
Confirmatory factor analysis for all items.

**Fig 3:**
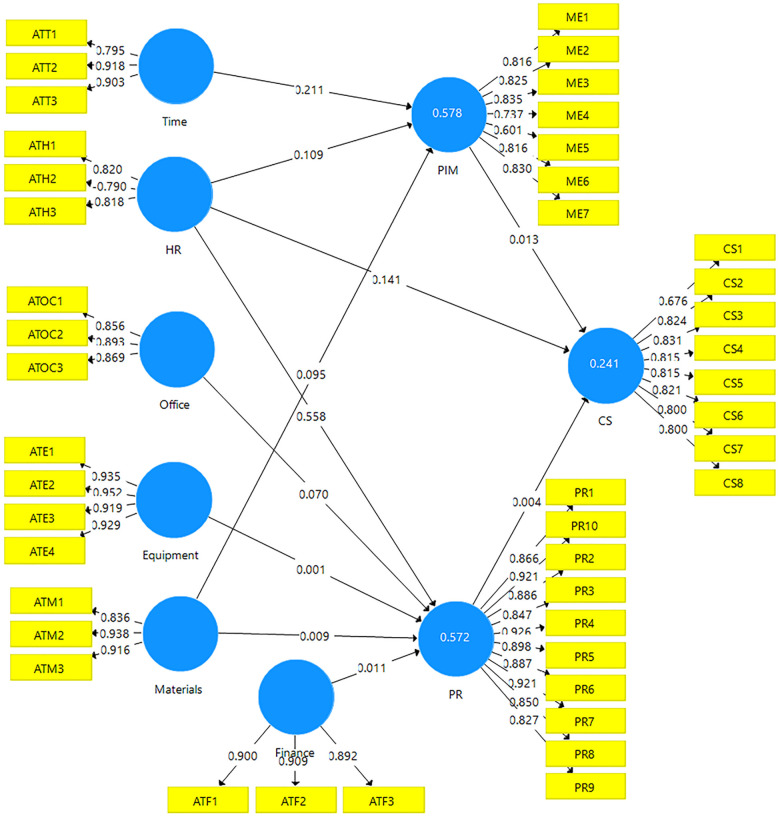
Confirmatory factor analysis for selected items. Source: Field survey (2022).

The PLS structural model was measured, and the results obtained are presented in Figs 2 and 3. In Fig 2, all items measuring every variable of the study were entered into the model. Thus, three items each were used to measure constructs such as time, materials, and finance, while four items each were used to measure equipment, office, and HR. Ten items constituted perceived relevance and, seven items constituted policy implementation, monitoring, and evaluation. Lastly, career success comprised eight items. It is clear that only HR and Office constructs had one item each scoring below the 0.60 threshold for the study and needed to be removed from the model.

Fig 3 revealed only items that loaded above the 0.60 threshold. The four original items measuring office and HR constructs in Fig 2 have been reduced to three items each for the same HR and office construct in Fig 3. Thus, all items for each variable in Fig 3 met the requirement as suggested by [20].

### Model’s internal consistency

The model’s internal consistency was ascertained with four indices: Cronbach’s alpha, rho_A, Composite Reliability, and Average Variance Extracted (AVE). The threshold suggested by experts [21] was that the first three indicators should record values not less than 0.70 thresholds. The minimum threshold required for the last indices (AVE) is 0.50 minimum threshold (20 Hair et al, 2021). Internal consistency results, as presented in Table 2, suggest that values obtained for Cronbach’s Alpha were between 0.843 and 0.969 with the exception of HR factors which recorded 0.387. All values obtained for the rho_A were between 0.737 and 0.973.m Values for the Composite Reliability also range from 0.906 and 0.965, except for the HR factor, which again recorded a value of 0.410. The AVE values also obtained for the internal consistency were also between 0.615 and 0.873. The HR factors were, however, maintained because it met the requirement under two of the indices (rho_A and Average Variance Extracted (AVE)), which were very stringent for this study as compared to the two other indices under which it performed below average (Cronbch’s Alpha and Composite Reliability). The results suggest that the model achieves reliability.

### Discriminant validity

Heterotrait-Monotrait Ratio (HTMT) was used to establish the discriminant validity, and the requirement of [21] was used to guide the assessment of the discriminant validity for the study. The results, as presented in Table 3, suggest that zero (0) was recorded for crosswise loading on the same construct and less than 0.85 between different constructs. It is clear from Table 3 that all the HTMT values obtained recorded 0 for the diagonal loading and values below 0.85 maximum thresholds for the attainment of discriminant validity. The results mean that the model attained a discriminant validity.

**Table 3:** Discriminant validity using Heterotrait-Monotrait Ratio (HTMT).

	CS	Equipment	Finance	HR	Materials	Office	PIM	PR	Time
CS	**0**								
Equipment	0.200	**0**							
Finance	0.256	0.662	**0**						
HR	0.549	0.371	0.443	**0**					
Materials	0.354	0.846	0.845	0.561	**0**				
Office	0.337	0.809	0.639	0.611	0.822	**0**			
PIM	0.325	0.564	0.777	0.634	0.742	0.610	**0**		
PR	0.323	0.399	0.420	0.827	0.475	0.601	0.609	**0**	
Time	0.286	0.707	0.816	0.459	0.773	0.689	0.773	0.452	**0**

Source: Field Survey (2022)

### Collinearity

[20] criterion of a maximum value of 3.30 was used to determine the presence of multicollinearity by comparing the values of the inner (VIF) Variance Inflated Factor (VIF) with the criterion, and the results are presented in Table 4. The results indicate that all the VIF values for all the variables of the study were below the 3.30 maximum threshold as recommended. The results, therefore, suggest that there was no presence of multicollinearity among the variables of the study in the model. Thus, further and high-level analysis could be carried out.

**Table 4:** Collinearity statistics using inner VIF values.

	CS	Equipment	Finance	HR	Materials	Office	PIM	PR	Time
CS									** **
Equipment								3.013	** **
Finance								2.354	** **
HR	2.099						1.274	1.417	** **
Materials							1.975	3.191	** **
Office								2.559	** **
PIM	1.539								** **
PR	2.295								** **
Time							1.792		** **

Source: Field Survey (2022)

#### Path relationship for hypotheses testing

Results presented in 5 of the hypotheses testing were in two folds. The first part examined the collective contribution of the model in explaining the variance in the dependent variable/s. Thus, the results show that the model explains about 0.241 variances in career success, 0.578 in policy implementation and monitoring, and 0.572 variances in the perceived relevance of guidance services. The second part of the results examines how the six determinants of the attitude of stakeholders related to the three main dependent variables for the hypotheses- perceived relevance (PR), policy implementation and monitoring (PIM), and career success (CS). The second part of the results in Table 5 indicates that three determinants of the attitude of stakeholders are significantly related to the perceived relevance of guidance services in Ghana. That is, financial provision for guidance services significantly relates to the perceived relevance of guidance service at (*β* = 0.104, *t* = 2.271, *p* = 0.024) for hypothesis two; availability of the competent human resource (HR) significantly relates to the perceived relevance of guidance service at (*β* = 0.582, *t* = 14.158, *p* = 0.000) for hypothesis three there was a significant relationship between dedicated office for guidance service and perceived relevance of guidance service (*β* = 0.277, *t* = 5.039, *p* = 0.000) for hypothesis five. Two determinants, however, failed to achieve a statistical significance with perceived relevance. That is office equipment had a non-significant relationship with the perceived relevance of guidance services at (*β* = 0.044, *t* = 0.748, *p* = 0.455) for hypothesis one; and materials had a non-significant relationship with the perceived relevance of guidance services in Ghana at (*β* = 0.126, *t* = 1.886, *p* = 0.060) for hypothesis four. Results for the hypotheses of the study have been presented in Table 5.

**Table 5:** Results for path analysis.

	R Square				R Square Adjusted			
**CS**	0.241				0.235			
**PIM**	0.578				0.575			
**PR**	0.572				0.566			
							**Confidence Intervals**	
	**Original Sample**	**Sample Mean**	**Standard Deviation**	**T Statistics**	**P Values**	**F^2^**	**2.5%**	**97.5%**
1. Equipment -> PR	0.044	0.041	0.058	0.748	0.455	0.001	-0.070	0.152
2. Finance -> PR	0.104	0.105	0.046	2.271	0.024	0.011	0.017	0.193
3. HR -> PR	0.582	0.586	0.041	14.158	0.000	0.558	0.666	0.502
4. Materials -> PR	0.126	0.126	0.067	1.886	0.060	0.009	0.259	0.010
5. Office -> PR	0.277	0.276	0.055	5.039	0.000	0.070	0.170	0.380
6. HR -> PIM	0.242	0.243	0.038	6.411	0.000	0.109	0.321	0.170
7. Time -> PIM	0.399	0.403	0.052	7.710	0.000	0.211	0.295	0.498
8. Materials -> PIM	0.281	0.280	0.052	5.388	0.000	0.095	0.175	0.371
9. HR -> CS	0.474	0.472	0.075	6.348	0.000	0.141	0.324	0.621
10. PIM -> CS	0.124	0.125	0.063	1.959	0.051	0.013	0.250	0.008
11. PR -> CS	0.081	0.080	0.094	0.863	0.389	0.004	-0.123	0.247

Source: Field Survey (2022)

All three determinants of attitude had a statistically significant relationship with policy implementation and monitoring (PIM). Human resource (HR) had a significant relationship with policy implementation and monitoring (PIM) at (*β* = 0.399, *t* = 1.886, *p* = 0.000) for hypothesis six; time allocation on teaching timetable for guidance service significantly related to PIM at (*β* = 6.411, *t* = 7.710, *p* = 0.000) for hypothesis seven; and Materials availability significantly influence PIM on guidance services in Ghana at (*β* = 0.281, *t* = 5.388, *p* = 0.000) for hypothesis eight. Human resource (HR) also significantly predicted career success (CS) at (*β* = 0.474, *t* = 6.348, *p* = 0.000). The last two hypotheses could not be supported because PIM had a non -statistically significant relationship with career success (CS) at (*β* = 0.124, *t* = 1.959, *p* = 0.051) for hypothesis ten, and perceived relevance also achieved a non-significant relationship with CS at (*β* = 0.081, *t* = 0.863, *p* = 0.389) for hypothesis eleven.

**Fig 4:**
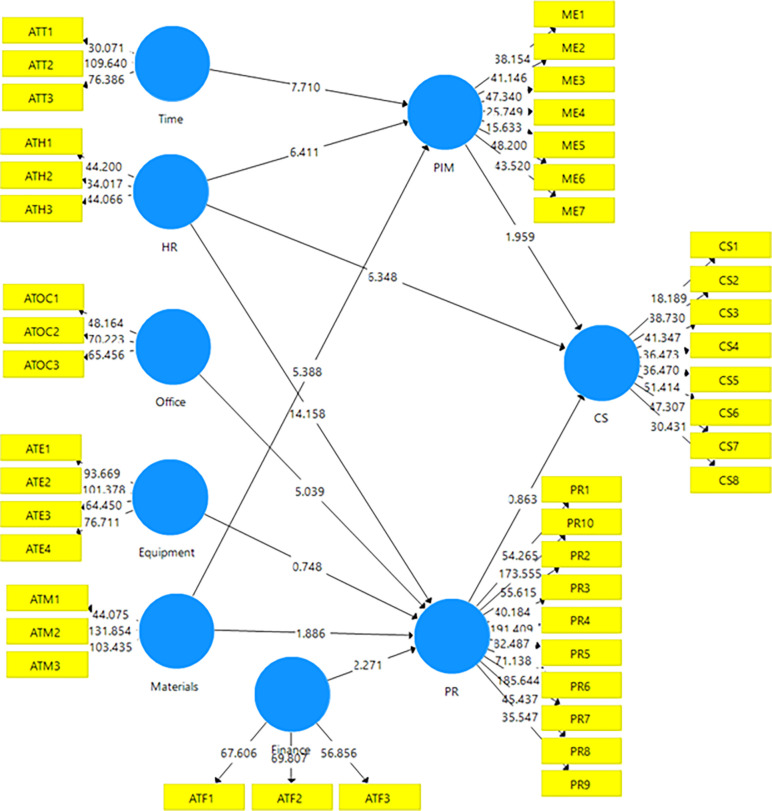
Bootstrapping. Source: Field survey (2022).

The effect sizes obtained for each of the significant paths reported in the model were based on [22] suggestion that an effect size of 0.010 to 0.401 was acceptable. The unidimensional nature of the confidence intervals for the variables for all significant paths also revealed valid and reliable significance. Additionally, the significant results were further strengthened by the confidence level of 97.5%, with a minor error margin of only 2.5% indicated by the statistics obtained from the upper and lower boundaries, respectively. The graphical representation of significant and non-significant relationships established for the hypotheses of the study presented in Table 5 can also be seen in Fig 4. Thus, Fig 4 confirms the results of the path relationship analysed using a bootstrapping sequence of 5000 samples to assess a structural model presentation, as [20] suggested.

## Discussion

The findings on perceived references of guidance services in Ghana among stakeholders have been found to be significantly and positively influenced by several dimensions of attitude in this study. The first determinant of attitude influencing the perceived relevance of guidance service was finance. The results suggest that guidance coordinators’ success largely depends on the availability of funds to purchase psychological tests, inventories, and stationery for guidance-related activities. It also means that in the absence of the funds, the guidance service coordinator might perform poorly. Also, expenses on guidance activities incurred by the coordinators needed to be reimbursed to enable them to continue to provide guidance-related services. For stakeholders to consider guidance services in Ghanaian basic schools as an integral component for the success of learners, the availability of funds is very important. The results corroborate the findings of [4, 11] that financial resources highly influenced the success of guidance services in schools.

Hypothesis three outlined the second determinant of stakeholders’ attitudes which was significantly related to the perceived relevance of competent human resources for guidance services at the basic schools in Ghana. This means competent and adequate human resources for guidance services at the basic schools will send a signal to other stakeholders that guidance services are very important. The results suggest that the background and qualification of people appointed as coordinators for guidance services should be scrutinized to ensure the appointment of the right coordinators. The results support the findings of [12] that adequate guidance coordinators with the requisite guidance skills are needed for successful guidance services.

A dedicated office for guidance services was the third determinant of stakeholders’ attitudes to have significantly influenced the perceived relevance of guidance services in Ghanaian schools. The results suggest that stakeholders will attach importance to guidance services and their delivery if there is a dedicated office for guidance services. A dedicated office for guidance services is also a panacea for ensuring confidentiality among parties. The results further suggest that pupils, guidance coordinators, and school authorities will give more credence and attach more seriousness to guidance services if separate office space exists for its activities. The results, therefore, agree with the findings of [15] lack of confidentiality service as a barrier to the patronage of guidance services.

However, two determinants of attitude–equipment, and materials- were found to have no statistically significant influence on the relevance attached to guidance services at the basic schools in Ghana. This means that though they might be important for guidance services, this study found that hypotheses one and four were not enough to influence the positive perception of guidance services among stakeholders. Therefore, the findings of this study disagreed with that of [3] who found that office equipment and materials influence the positive perception of guidance services among learners.

The three determinants of attitude that influence policy implementation and monitoring (PIM) as found in hypotheses six, seven, and eight, were human resource, materials, and time, respectively. The results revealed that policy implementation regarding guidance services at the basic school level is strongly premised on the availability of adequate and competent human resource. Guidance service providers should be knowledgeable and hold the requisite qualifications to be able to implement and monitor policies on guidance services at the basic school level. Thus, the assertion by [2] that competent and adequate human resource is needed for a successful guidance service holds for the findings of this study. Also, the findings further suggest that it is not enough to have adequate and competent human resources, rather, the availability of material resources to be used for successful policy implementation and monitoring of guidance services. Though material resources were found not to have influence on the perceived relevance of guidance services, they are rather important in influencing policy implementation and monitoring at the basic school level. The findings, therefore, are in tandem with the findings of [13] that the availability of material resources or logistics enhances the chances of implementing policies. Furthermore, time allocation for guidance services on the teaching timetable was also found to have influenced policy implementation on guidance services at the basic school level in Ghana. The findings of this study further corroborate the earlier findings of [10] that lack of time allocation for guidance services at the basic school level affects the successful implementation of guidance services.

The only determinant of attitude that has the potential to influence career success is, adequate and competent human resources, as established in hypothesis nine. Human resource was not only found to have a significant relationship with PIM and the perceived relevance of guidance services but also with career success. The results suggest that quality human resources influence effective outcomes of guidance services at the basic school level in Ghana. The results agreed with earlier findings by [2] that competent guidance coordinators are required to ensure that guidance services positively impact the beneficiaries.

Perceived relevance (PR) and policy implementation and monitoring (PIM) failed to significantly influence career success. The results mean that PR and PIM do not directly relate to career success or were not enough for predicting career success. Thus, there is a need for other mediation variables not considered in the model to ensure that PR and PIM lead to career success. The results confirm that of [14] that PIM had no significant relationship with career success. The results further disagreed with the findings of [13] who rather found a significant relationship between perceived relevance and career success.

### Theoretical and practical implications

The theoretical and practical implication of the findings of this study is that there are determinants with respect to the attitude of stakeholders that influence both perceived relevance, policy implementation and monitoring, and career success. Among all the determinants of attitude considered in this study, competent and adequate human resource was found to be the only determinant that influenced the three dimensions (CS, PIM, PR) considered in this study. Addressing human resource needs for guidance services will ensure effective guidance services at the basic school level. Therefore, to ensure that policies on guidance services are implemented and monitored leading to career success, the government and heads of basic schools should appoint competent and adequate human resources to undertake guidance services at the basic school level. Other determinants that required the attention of heads of basic schools and practical government attention were time, material resources, and dedicated office space for guidance services.

## Conclusion and recommendations

This study examines determinants of stakeholders’ attitudes that influence policy implementation and monitoring, perceived relevance, and career success. It can be concluded that the availability of funds (finance) for payment of expenses incurred in relation to guidance services, competent and adequate guidance coordinators (HR), and dedicated office to ensure confidentiality for guidance services were the three determinants that predicted the perceived relevance of guidance services in Ghana. Additionally, provisions for guidance services on the teaching timetable, availability of logistics (Material resources), and quality guidance coordinators (HR) were the only three determinants that influenced policy implementation and monitoring of guidance services at the basic school levels in Ghana. Human resources (HR) again emerged as the only determinant that influenced the career success of beneficiaries in Ghana.

It was therefore recommended that Ghana Education Service and heads of basic schools should appoint quality human resource guidance coordinators for guidance services at the basic school level. Requisite academic qualifications in guidance and counselling, knowledge of guidance services, and an appropriate number of guidance coordinators depending on the number of pupils in every basic school should be considered when appointing the coordinators. It is also recommended that the Ghana Education Service and heads of basic schools should ensure that guidance services are allocated time on the timetable, and logistics/material resources should be provided to enable guidance coordinators to deliver on their mandates. Finally, Ghana Education Service and heads of basic schools should provide funds for payment of guidance service-related expenses. The fund could be established at the regional, district, and school levels to ensure that monitoring of guidance policies is effectively executed.

### Limitations suggestion for further studies

The findings of this study are limited to determinants of attitude on the perceived relevance of guidance services, career success, and policy implementation and monitoring at the basic school level in Ghana. The stakeholders in this study largely were regional, district, and school-level counsellors and heads of basic schools in Ghana. The actual beneficiaries’ views on the issues interrogated were missing. It is therefore suggested that further studies should focus on the perspectives of beneficiaries of guidance services and the impact of such services on their career success.

## References

[1] Kanga BM. Effectiveness of guidance and counselling services in enhancing students’ adjustment to school academic environment in public boarding secondary schools in Kenya. International Journal for Innovation Education and Research. 2017 July; 5(7): 75–87

[2] Asiedu-Yirenkyi C, Kyere EA, Ofori KN. Evaluation of guidance and counselling practices in schools: A case study of Manhyia sub-metropolis, Ghana. International Journal of Education. 2019 Mar;4(28):52–63.

[3] Andegiorgis GE. Status of counselling services in secondary schools in Keren sub-zone, Anseba Region, Eritrea. Global Journal of Guidance and Counseling in Schools: Current Perspectives. 2019;9(1):048–55.

[4] Letsa B, Sadiq A. Challenges facing guidance and counselling coordinators in Senior High Schools in the Kumasi Metropolis in Ghana. International Conference on Applied Science and Technology Conference Proceedings 2021 Apr 26 (Vol. 6, No. 1).

[5] Dankyi LA, Minadzi VM, Segbenya M, Agyei PM, Dankyi JK. Examining stakeholders’ perception of sixty-six years of guidance service delivery in Ghana: The explanatory sequential mixed method perspectives. Cogent Social Sciences. 2024 April 10:1, 2337900,

[6] Hooley T, Dodd V. The economic benefits of career guidance. 2015

[7] Hanimoglu E. The perceptions of students about the role of school counselors on career selection. European Journal of Educational Research. 2018;7(4):763–74.

[8] Dankyi LA, Minadzi VM, Segbenya M, Agyei PM, Dankyi JK. Examining stakeholders’ perception of sixty-years of guidance service delivery in Ghana. The explanatory sequential mixed method perspectives. Cogent Social Sciences. 2024; 10(1): 2337900

[9] Oluwatosin SA. Stakeholders’ perception of school guidance and counselling services effectiveness in Ekiti State Southwestern, Nigeria. Asia Pacific Journal of Education, Arts and Sciences. 2016 Oct;3(4):72–5.

[10] Musorewa NA., Ngunjiri M, Mukadi EB. The influence of the availability of facilities and resources in the effective provision of guidance and counselling services in secondary schools in Gesusu Ward, Aasaba South Sub-County, Kisii County, Kenya. International Journal of Advance Research. 2018; 6(10): 573–577.

[11] Egbo AC. The challenges of guidance and counselling practices as perceived by secondary school counsellors in Enugu State Nigeria. International Journal of Education and Research. 2015 May;3(5):375–84.

[12] Uba MB, Eze EN, Elom C. Challenges facing effective implementation of guidance and counseling services in secondary schools in Oyi Local Government Area of Anambra State. Webology (ISSN: 1735-188X). 2022;19(3).

[13] Ndanu NR, Mukadi EB, Tarus PK. Effectiveness of guidance and counseling programme in enhancing students’ retention in public day secondary schools in Nyahururu sub-county, Kenya. European Journal of Education Studies. 2022 Mar 3;9(2).

[14] Olanrewaju MK, Suleiman Y. Perception assessment of guidance and counseling services among educational stakeholders in selected secondary schools in Oyo State, Nigeria. Indonesian Journal of Educational Counseling. 2019 Jan 31;3(1):31–42.

[15] Boitt ML. Evaluation of the implementation of guidance and counselling programme aspects in Baringo County extra secondary schools, Kenya (Doctoral dissertation, Egerton University).

[16] Sampson JP Jr, Bullock-Yowell E, Dozier VC, Osborn DS, Lenz JG. Integrating Theory, Research, and Practice in Vocational Psychology: Current Status and Future Directions. Online Submission. 2017. doi: 10.17125/svp2016

[17] Bandura A. Self-regulation of motivation and action through internal standards and goal system. Goal concepts in personality and social psychology. 1986:19–85.

[18] Krejcie RV, Morgan DW. Determining sample size for research activities. Educational and psychological measurement. 1970 Sep;30(3):607–10. doi: 10.1177/001316447003000308

[19] Pallant J. A step-by-step guide to data analysis using the SPSS program. 2010.

[20] Hair JF Jr, Hult GT, Ringle CM, Sarstedt M, Danks NP, Ray S. Partial least squares structural equation modeling (PLS-SEM) using R: A workbook. Springer Nature; 2021.

[21] Henseler J, Ringle CM, Sarstedt M. A new criterion for assessing discriminant validity in variance-based structural equation modeling. Journal of the academy of marketing science. 2015 Jan; 43:115–35.

[22] Cohen J. Statistical power for the behavioral sciences. Hilsdale. NY: Lawrence Erlbaum. 1988;58(1):7–19.

